# Label-free detection of hypoxia-induced extracellular vesicle secretion from MCF-7 cells

**DOI:** 10.1038/s41598-018-27203-9

**Published:** 2018-06-20

**Authors:** Tugba Kilic, Ana Teresa De Sousa Valinhas, Ivan Wall, Philippe Renaud, Sandro Carrara

**Affiliations:** 1 Swiss Federal Institute of Technology Lausanne, EPFL, Integrated Systems Laboratory (LSI), 1015 Lausanne, Switzerland; 2 Swiss Federal Institute of Technology Lausanne, EPFL, Microsystems Laboratory 4 (LMIS4), 1015 Lausanne, Switzerland; 3 Department of Biochemical Engineering, University College London, Bernard Katz Building, Gordon Street, WC1H 0AH London, England

## Abstract

Nanoscale extracellular vesicles (EVs) including exosomes (50–150 nm membrane particles) have emerged as promising cancer biomarkers due to the carried genetic information about the parental cells. However the sensitive detection of these vesicles remains a challenge. Here we present a label-free electrochemical sensor to measure the EVs secretion levels of hypoxic and normoxic MCF-7 cells. The sensor design includes two consecutive steps; i) Au electrode surface functionalization for anti-CD81 Antibody and ii) EVs capture. The label-free detection of EVs was done via Differential Pulse Voltammetry (DPV) and Electrochemical Impedance Spectroscopy (EIS). The working linear range for the sensor was 10^2^–10^9^ EVs/ml with an LOD 77 EVs/mL and 379 EVs/ml for EIS and DPV based detection. A blood-abundant protein, RhD was used for the selectivity test. In order to assess the performance of the biosensor, the level of EVs secretion by the human breast cancer MCF-7 cell line was compared with enzyme-linked immunosorbent assays (ELISA) and Nanoparticle Tracking Analysis (NTA). Designed label-free electrochemical sensors utilized for quantification of EVs secretion enhancement due to CoCl_2_-induced hypoxia and 1.23 fold increase with respect to normoxic conditions was found.

## Introduction

Extracellular vesicles (EVs) are widely recognised due to their significant contribution to intercellular communication via protein, lipid and RNA transport from parent cell to recipient cell^1,2^. The classification and nomenclature of EVs are still being defined due to ongoing debate regarding biogenesis and associated biological processes^1–4^. EVs are usually characterized and quantified by well-known exosomal biomarkers such as tetraspanins CD-9, CD-64, CD-81, CD-53, CD-37 and cytosolic proteins of Tsg101, Alix, or cytoskeletal proteins^1–4^. In addition to those characteristic protein cargos, EVs also carry genetic information via DNA, coding/non-coding RNA like miRNAs^5^.

Paramount amount of research showed the potential use of EVs in the clinic due to their antitumoral immune response stimulation, induction of tolerogenic effects and involvement in metastatic processes^6,7^. In addition to that, EVs are present in most bodily fluids, therefore are considered as non-invasive biomarkers for early cancer detection and monitoring treatment efficacy^8^ which are present in blood in concentrations ranging from 10^8^ to 10^11^ EVs/ml^9^.

Breast cancer, one of the leading causes for malignancies in women, has recently been associated with EVs due to EVs -mediated tumor angiogenesis stimulation, drug resistance promotion and re-establishment of tumour microenvironment via reorganization of stroma^10^. In addition to those biological functions, it is reported that, hypoxia-induced EVs release could be one of the reasons for malignant transformation followed by proliferation and migration, since enhanced HIF-1α (Hypoxia-inducible factor 1-alpha) shown to increase EVs release and resulted in an aggresive cell phenotype^11^. Therefore, EVs hold great promise as non-invasive biomarkers for breast cancer diagnosis, progression and monitoring treatment efficiency^8,10^. In addition to this, EVs will also play a role in the creation and verification of in *in vitro* models of cancer, aiding in the development of therapeutical drugs. This highlights the importance of detecting EVs from conditioned medium. However, isolation and quantification of EVs are still challenging. Ultrancentrifugation has been accepted as the golden standard for purification and isolation of EVs whereas western blot analysis and enzyme-linked immunosorbent assays (ELISA) have been used for analysis and detection purposes. Drawbacks associated with these techniques such as time consumption, large sample volume requirement and labelling steps necessitates novel techniques for easy, label-free and sensitive EVs detection and analysis^12^.

Up to now, various sensing technologies have been developed for detection and profiling of exosomes^13^. Plasmonic sensing systems based on surface plasmon resonance (SPR)^14–16^ have been shown to provide label-free sensing schemes with minimal sample volume as low as 0.3 μl. Electrochemical sensors offer advantages in EVs sensing due to their miniaturization capability, affordable cost and high detection limits^17–22^. Most of the sensors developed so far provided a proof-of-concept for EVs sensing with defined limit of detection (LOD), with the exception of a few, which have been applied to analyse clinical samples or study a biological question^16,18,23,24^. However, considering the final aim of these biosensors, it is crucial to test their performance for a specific biological context.

With this work, for the first time in literature, we aim to demonstrate a label-free, electrochemical biosensor that is able to detect the increased EVs release from breast cancer cell line, MCF-7 due to CoCl_2_ induced hypoxia^11^. The principle behind the biosensor involves the monitoring of changes in electrochemical signals due to bio recognition reaction between anti-CD81 antibody and CD-81 present on the lipid membrane of breast-cancer EVs. The designed disposable, simple EVs biosensor provides the best detection limit (LOD) of 77 EVs/ml to the best of our knowledge among others reported in literature and has the dynamic detection range of 10^2^–10^9^ EVs/ml. Thanks to its lower LOD compared to any other electrochemical EVs biosensors in literature, and its selectivity against CD-81 tested with one of the most abundant blood protein, RhD, the proposed biosensor holds great potential for not only detection of EVs from blood samples but also for integration with platforms that mimic tumor microenvironment for chemotherapeutic drug testing.

## Methods

### Chemicals and reagents used

MCF-7 cell line was purchased from ATCC ATCC. RPMI 1640 + GlutaMAX^TM^, Fetal Bow Serum (FBS), Penicillin/Streptomycin (Gibco- 151140122) and insulin (Gibco 12585-014) were bought from Gibco, Life Technolgies. Immunostaining reagents; HIF-1 α antibody, 2-(4-amidinophenyl)-1H -indole-6-carboxamidine (DAPI, D21490) was purchased from Invitrogen, Alexafluor 488 were provided by histology core facility in Life Sciences department of EPFL. Cobalt chloride, CoCl_2_ (C8661) and other chemicals were purchased from Sigma. Gold screen-printed electrodes (Au SPE) used throughout the study were bought from Dropsens.

### Electrochemical Measurements

Measurements of EIS/DPV were performed using an electrochemical working station (Metrohm Autolab PGSTAT 302 N with Nova 1.11 software) with a conventional three-electrode system. For EIS, the potential was set to 0.100 mV and the frequency was scanned in a range of 10^−1^ to 10^5^ kHz at a 0.005 mV of AC amplitude. For DPV measurements, potential was scanned from −0.2 V to + 0.5 V with a step potential of 0.002 V/s. For both DPV and EIS measurements, an equimolar solution of 5 mM K_3_[Fe(CN)_6_]/K_4_[Fe(CN)_6_] in 0.1 M KCl was used for all electrochemical measurements.

### Immobilization Process for Antibody Biosensor

Electrodes were cleaned with PBS and ethanol prior to their use. Afterwards Au SPEs were immersed in a solution of 10 mM of 11-marcaptoundenoic acid (11-MUA) for 1 hour at room temperature to generate a thiol terminated self assembly monolayer (SAM). After washing the surface with ethanol and PBS, the electrodes were incubated in a EDC (50 mM)/NHS (50 mM) solution for 30 minutes at room temperature. After this step, the Au SPE surface was exposed to 600 μg/mL of neutravidin (Thermofisher Scientific) for 1 hour at 4 °C. Then, 5.5 ug/mL of biotinylated anti-CD81 antibody (Miltenyi Biotec) solution was incubated on the electrodes for 16 hours at 4 °C.

### Cell Culture and Treatment

MCF-7 cells were maintained in RPMI 1640 + GlutaMAX^TM^ supplemented with 10% HI FBS, 1% Penicillin/Streptomycin and 10 μg/mL of human recombinant insulin (ThermoFisher Scientific) in a standard culture incubator with humidified air containing 5% CO_2_ at 37 °C. For EVs studies, FBS free of EVs was used. EVs free FBS was prepared through an 18 hours ultracentrifugation at 4 °C and 100 000 × g. Cells were seeded at 1.5 × 10^6^ per well in 6-well plates in the day before the experiment. The next day, medium containing 200 μM of CoCl_2_ was added to the cultures, using normal medium as control. Cell counting was performed with a haemocytometer and viability was assessed with the trypan blue exclusion method.

### EVs Isolation and Purification

EVs isolation was performed as previously described^25^. Briefly, cell culture supernatants were centrifuged at 300 × g for 5 minutes. The supernatants were transferred to a clear tube and centrifuged at 2000 × g for 10 minutes, followed by centrifugation at 10 000 × g for 30 minutes. The supernatants were passed through a 0.22 μm filter and were ultracentrifuged for 2 hours at 100 000 × g. After discarding the supernatant, the pellets were resuspended in PBS and ultracentrifuged for 2 hours at 100 000 × g. The pellet was resuspended in 1 mL of PBS and stored at 4 °C for analysis performed within a week or −20 °C for long-term storage.

### EVs Nanoparticle Tracking Analysis (NTA)

The analysis of absolute particle distribution was performed using the NanoSight NS300 (Malvern Panalytical). The NTA measurement conditions were: temperature = 22 °C; viscosity = 0.95 cP; frames per second = 21, measurement time = 91 s. Three records were performed per sample.

### Immunocytochemical Assay

MCF-7 grown on 96 well-plates were fixed with 4% paraformaldehyde in PBS for 20 min at room temperature, washed with PBS, permeabilized with 0.1% Triton X-100, washed with PBS and blocked with 1% bovine serum albumin in PBS. Cells were incubated overnight with anti HIF1-α antibody (Abcam) at 1:200 dilution, washed with PBS, and incubated for 1 hour at room temperature with secondary antibody at 1:200 dilution. Samples were washed with PBS and counter stained with DAPI. Fluorescent images were obtained with a Zeiss LSM700 confocal microscope and processed using ImageJ.

### Enzyme-Linked Immunoabsorbant Assay

An ELISA kit for CD-81 Ab based detection of EVs was purchased from System Biosciences. After purification of EVs as decribed above, samples were processed according to manufacturer’s instructions.

### Statistical Analysis

All data were obtained from three independent experiments, and expressed as means** ± **SD. One-way ANOVA was used to compare data from different experimental groups and the difference between them was estimated by the least significant difference (LSD) test. p < 0.05 was considered statistically significant.

## Results

**Figure 1:**
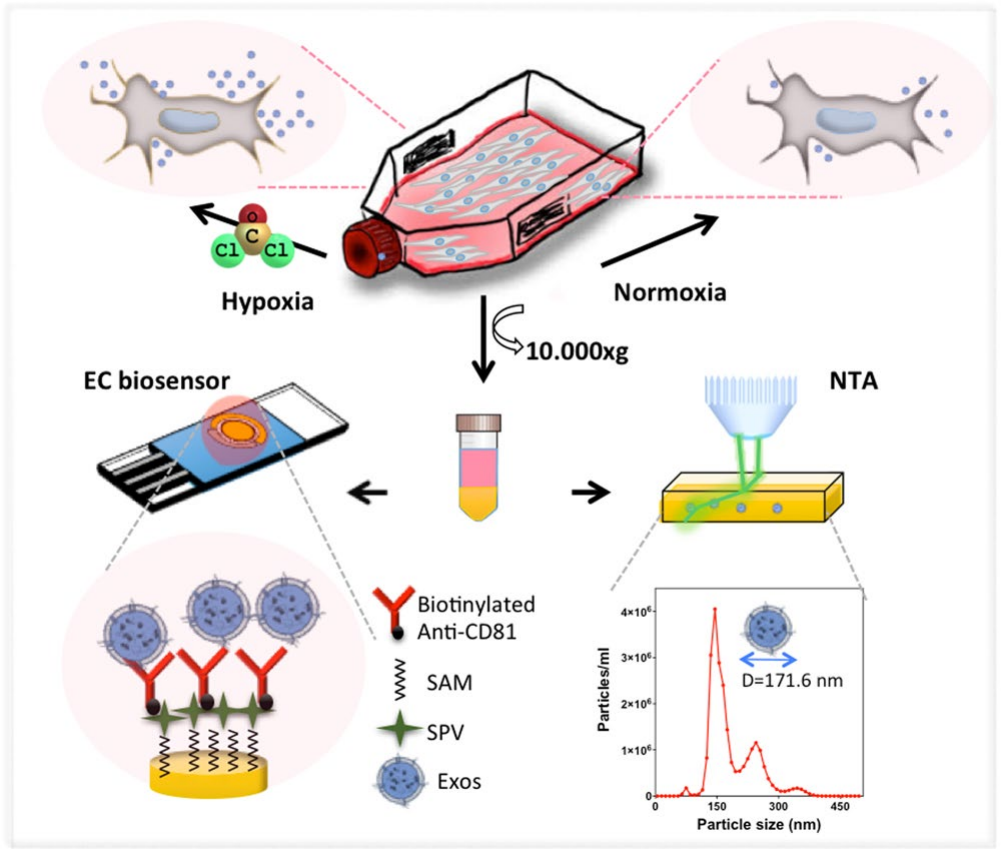
Experimental steps followed throughout the work. MCF-7 cells were exposed to either CoCl_2_- induced hypoxic or normoxic conditions. Isolation of EVs were done via ultracentrifugation. Characterization and quantification of EVs were done via NTA and EVs biosensors that are designed to capture CD-81 EVs biomarker via biotinylatd anti-CD81 antibody immobilized through streptavidin-biotin interaction on SAM modified Au SPE surface.

The conceptual drawing of the experimental steps was depicted in Fig. 1. The effect of hypoxia/normoxia conditions on EVs secretion level was investigated by label-free electrochemical biosensors upon detection of CD-81 EVs surface biomarker via DPV and EIS methods. These two approaches were used since both methods have advantages and disadvantages over each other, such as being fast as in the case of DPV but having the compromise between being less destructive and taking longer time for the measurement for EIS. Prior to detection, EVs were isolated from cell culture supernatant via ultracentrifugation as detailed in *section 2*.*2* and characterized via NTA in terms of concentration (number of EVs/ml) and size. Experimental results regarding label-free detection of EVs at different culturing conditions are discussed in the following section.

### Isolation of EVs from MCF-7 cell line

**Figure 2:**
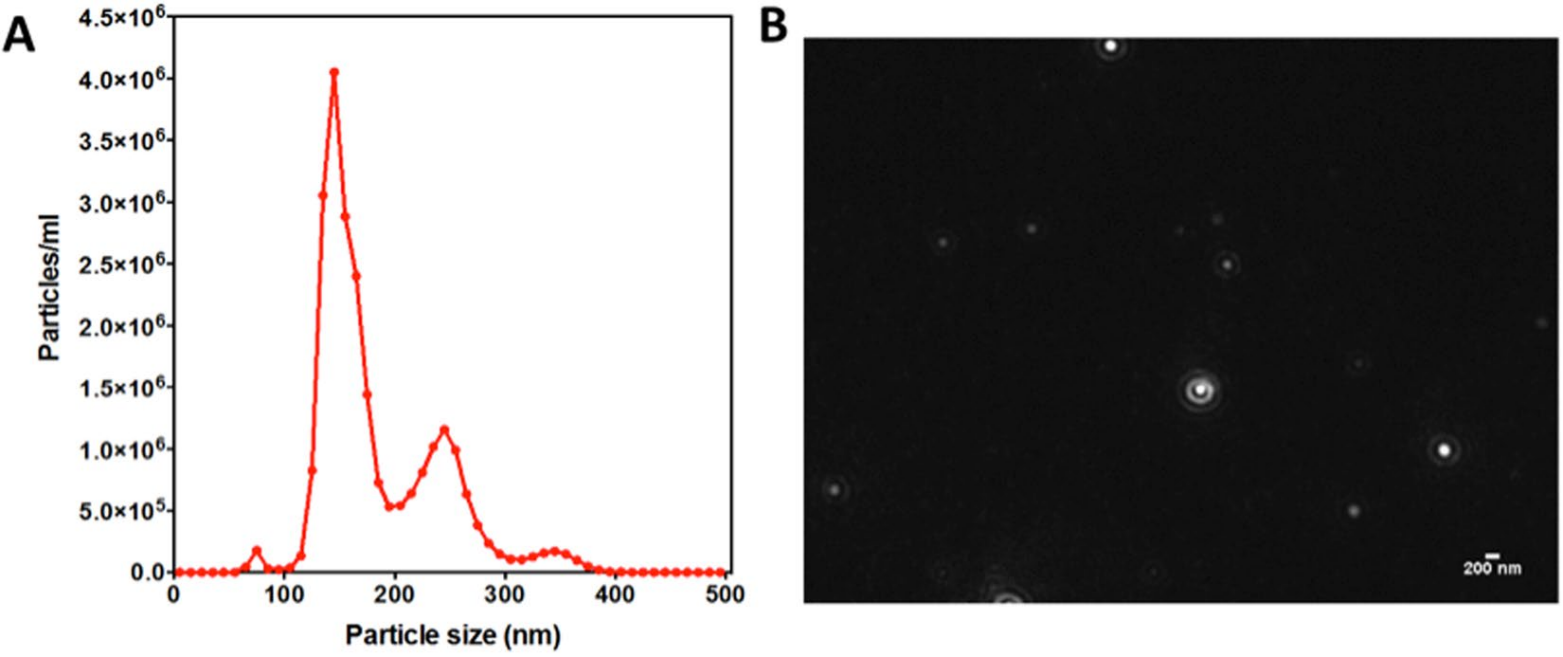
Characterization of EVs via NTA. Size distrubution analysis of EVs as a function of particle concentration. (**A**) Representative image used for size distribution analysis of extracellular vesicles (scale bar represents 200 nm).

The isolation of cancerous EVs was performed through ultracentrifugation as described in methods section. Nanoparticle tracking analysis measurements gave a size distribution with a mean diameter of 171.6** ± **51.7 nm, in the expected size range as can be seen in Fig. 2A. Considering the two peaks in the NTA graph, and mean diameter of 171.6 nm, our sample contain heteregoneous mixture of exosome and microvesicles including ectosomes, membrane particles and apoptotic vesicles as a typical result of high-speed ultracentrifugation protocols reported elsewhere^26,27^. Therefore, as other biosensor papers in literature^28,29^, in this work we will use EVs as a more general term. Similar to other optical^30^ and electrochemical biosensors^17,24^ designed for EVs detection, in our work we assumed that the EVs suspensions are monodisperse with a mean diameter, obtained from the NTA analysis. Figure 2B shows the real time captured image of NTA analysis where EVs seem as white circles.

### Electrode functionalization with biotinylated anti-CD81 antibody

**Figure 3:**
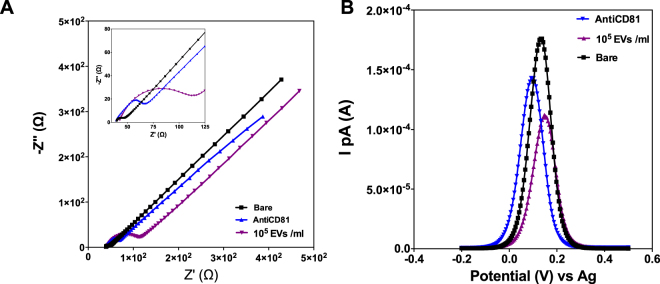
Electrochemical characterization of the surface functionalization based on EIS (**A**) and DPV (**B**) according to the steps detailed in section 2.3.

As a first step of biosensor development, the commercially available gold screen printed electrode (Au-SPE) surface was functionalized for biotinylated anti-CD81 capture. To do that, self-assembly monolayer (SAM) was immobilized on the sensor surface via immobilization of 11-mercaptoundodecanoicacid followed by N-ethylcarbodiimide (EDC)/ N-hydroxysuccinimide (NHS) interaction for neutravidin capture^31,32^. Neutravidin has been chosen due to its key features as being carbohydrate free, neutral isoelectric point that minimizes non-specific binding. After neutravidin immobilization, biotinylated anti-CD81 was incubated on the electrode surface for the strong covalent binding between biotin and neutravidin. Characterization of the electrode surface upon each functionalization step was done by EIS and DPV. The complex impedance data recorded by EIS measurements were presented by Nyquist plots whereas voltammetric measurements were presented in differential pulse voltammograms, as can be seen in Fig. 3A,B. As typical Nyquist plots modelled according to Randles’ circuit which is composed of solution resistance (R_s_), charge transfer resistance (R_ct_) and double-layer capacitance (C_dl_), Fig. 3A shows the change in the imaginary component of impedance (|−Z″|, capacitance based) as a function of the real component (Z′, resistance based) of impedance with respect to different functionalization steps. The R_ct_ value that corresponds to the diameter of semi-circular Nyquist plots increased after each functionalization step due to increased blockage of the redox probe, Fe(CN)_6_]^3/4^ through AuSPE surface. Expectedly, anodic current of redox probe (IpA) decreases upon successive immobilization of biotinylated anti CD81-Ab and EVs as shown by the differential pulse voltammogram represented in Fig. 3B.

### Sensitivity of the biosensor

Having seen the successful functionalization of the sensor surface, we have tested various CD-81 marked MCF-7 EVs concentrations (1 × 10^1^ to 10^10^ EVs/mL) to determine the sensitivity of the biosensor. Altough the detection of EVs was achieved in a concentration-dependent manner based on capturing surface exposed CD-81 proteins, it is very important to ensure uniform size distribution for EVs since electrode surface saturation would be affected depending on the size of vesicles. To ensure uniform size distribution, exosome solutions were mixed gently before its use.

**Figure 4:**
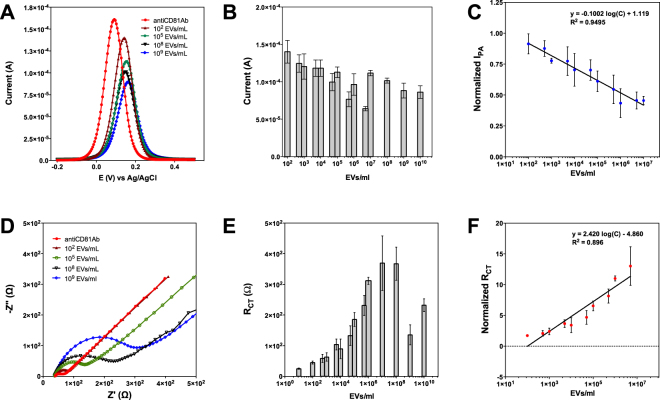
Assessment of biosensor sensitivity via concentration studies. Differential pulse voltammograms recorded for various EVs concentrations (10–10^10^ EVs/ml) (**A**), bar graphs represent the average Ip_A_ of redox probe at each concentration, (**B**) semi-log calibration curve drawn for DPV based detection of EVs in the concentration range of 10^2^–10^7^ EVs/ml (**C**). EIS measurements in the concentration range of 10^2^–5 × 10^6^ EVs/ml (**D**), bar graphs with represent the average R_ct_ of redox probe at each concentration (**E**) Semi-log calibration curve drawn for EIS based detection of EVs (**F**). Data are expressed as mean ± SD of three measurement. Experimental steps are detailed in methods section.

EVs are negatively charged in hydrophilic buffers^33^. The peak shift to more positive values with respect to increasing exosome concentration (Fig. 4A) is attributed to the fact that the electron transfer becomes slower due to decreased diffusion coefficient and hence a greater potential is required to overcome the kinetic barrier. Similarly, due to steric hindrance of EVs, access of Fe(CN)_6_]^3/4^ to electrode surface become more difficult and as a result, oxidation peak current decreases. A similar approach was used by Shiddiky *et al*.^19^ who compared the exosome secretion levels of normal and cancerous cells based on DPV signal of Fe(CN)_6_]^3/4^.

Calibration curves (Fig. 4C,F) were drawn according to normalized R_ct_ values (Normalized R_ct_ = (R_ct_)_EVs_/(R_ct_)_Ab_) derived from EIS data (Fig. 4D) and normalized Ip_A_ values (Normalized Ip_A_ = (Ip_A_)_EVs_/(Ip_A_)_Ab_) recorded by DPV data (Fig. 4A). The reason of using normalized data is the change in bare electrode R_ct_ and Ip_A_ values due to variability in surfaces from electrode to electrode. Concentration signal curves were fitted to calibration lines and the linear regression equation for analysing EVs in MCF-7 cell line was found to be

1 $${{\rm{Normalized}}{\rm{Ip}}}_{{\rm{A}}}=-\,0.1002\,\mathrm{log}(\mathrm{EVs}\,\mathrm{concentration})+1.119$$

for DPV and

2 $${\rm{N}}{\rm{o}}{\rm{r}}{\rm{m}}{\rm{a}}{\rm{l}}{\rm{i}}{\rm{z}}{\rm{e}}{\rm{d}}\,{{\rm{R}}}_{{\rm{c}}{\rm{t}}}{\rm{=}}2.420\,{\rm{l}}{\rm{o}}{\rm{g}}({\rm{E}}{\rm{V}}{\rm{s}}\,{\rm{c}}{\rm{o}}{\rm{n}}{\rm{c}}{\rm{e}}{\rm{n}}{\rm{t}}{\rm{r}}{\rm{a}}{\rm{t}}{\rm{i}}{\rm{o}}{\rm{n}})-4.860$$

for EIS measurements with a correlation coefficient (R^2^) of R^2^ = 0.95 and 0.90 respectively. The linear dynamic range for both methods was found to be 10^2^−10^9^ EVs/ml. The relative standard deviation (%RSD) of the measurements showed good reproducibility (<9.6%) using three measurements. The limit of detection (LOD) was calculated as 77 EVs/mL and 379 EVs/ml for EIS and DPV based detection, respectively. LOD was calculated according to the formula^34^

3 $${{\rm{y}}}_{{\rm{L}}{\rm{O}}{\rm{D}}}={{\rm{y}}}_{{\rm{b}}{\rm{l}}{\rm{a}}n{\rm{k}}}+3{\rm{S}}{{\rm{D}}}_{{\rm{b}}l{\rm{a}}{\rm{n}}{\rm{k}}}$$

where the average normalized signals of the lowest EVs concentration was used as y_blank_ and the SD of the measurement was used as SD_blank_.

These findings clearly show that, the designed label-free electrochemical biosensor is very sensitive for detecting EVs considering the reported concentration of EVs in cancer patient plasma as 5.6 × 10^10^ EVs/ml^35^. Moreover, our biosensor has achieved the best sensitivity compared to recently reported electrochemical^17,20,21,24^, optical^16,18,23^ and other conventional methods such as western blot^36^ (see Table 1). In addition to the lowest LOD, our method is label-free as contrary to other methods that require enzyme labels such as TMB^20,22^ and requires neither expensive equipment nor laborious experimental steps. In addition, we have used  low cost, disposable screen printed electrodes that provided reproducible results without any need for expensive microfabrication procedures.

**Table 1:** List of reported EVs biosensor platforms.

Sample	Target antibody	Method	Detection range, LOD	Reference
Ascites samples from ovarian cancer patients	CD24 and EpCAM	Nanoplasmonic detection based on transmission spectral shifts	1 × 10^7^ EVs/ml (3,000 EVs in 0.3 μl of sample per channel/marker)	^16^
Ovarian cancer patient plasma	CA-125, EpCAM, CD24	Fluorescence CCD imaging	7.5 × 10^5^ particles/ml	^39^
hepatoma cell lines MHCC97H/L and mouse melanoma cell lines B16-F1/10	CD9, CD41b and MET	SPR imaging	not mentioned	^15^
HepG2 cell line	CD63	SPR analysis and electrochemical detection of redox label	1 × 10^6^ particles/ml	^17^
MCF-7 cell line	CD9	Amperometric detection based on enzymatic conversion of TMB	2 × 10^5^ particles/ml	^20^
Embryonic kidney HEK293 cell lines	CD81	EIS	1.9 × 10^5^ particles ml	^21^
MCF-7 cell line	CD63	H_2_O_2_-mediated oxidation of TMB	5.2 × 10^5^ particles/ml	^22^
BT474 and MDA-MB-231 cell line	CD9, CD63 and HER2	SPR	3.3 × 10^7^ particles/ml	^40^
MCF-7 cell line	CD81	Label-free detection via DPV and EIS	77 particles/ml for DPV and 379 particles for EIS	*This work*

### ELISA measurements

**Figure 5:**
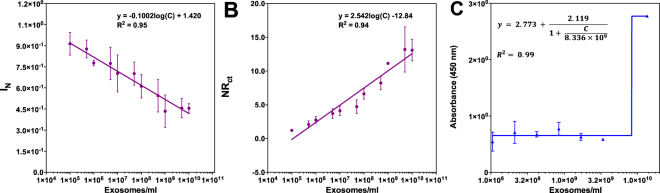
Calibration curves drawn by ELISA standards based on DPV (**A**) EIS (**B**) and ELISA (**C**). Data are expressed as mean ± SD of three measurements. Experimental steps are detailed in methods section.

In order to compare the LOD of the biosensor, we have performed ELISA measurements Calibration curves for ELISA measurements were fitted to Sigmoidal, 4 parameter logistic regression (4PL), where X is the log(EVs concentration) (Fig. 5). The limit of detection (LOD) of ELISA was calculated as 8.34 × 10^9^ EVs/ml according to the method previously used to calculate the LOD of the developed sensors.

Electrochemical measurements have been repeated with ELISA standards to show that, the standards, i.e. CD81 antigen, do not give the same response as the EVs and would lead to a wrong interpretation. This would make it incorrect to quantify the amount of EVs through the calibration curve drawn by standard samples. As can be seen in Fig. 5A,C, calibration curve equations of DPV and EIS based detection are different than the ones represented in Fig. 4C,F respectively. As a result, calculated limit of detection (LOD) for the biosensor has varied from 1.44 × 10^3^ EVs/ml to 77 EVs/mL for DPV technique considering standard and EVs samples respectively. On the other hand, LOD was calculated for EIS method as 3.96 × 10^5^ EVs/ml for standard and 379 EVs/ml for EVs samples. We think that the reason to this abrupt change between standard and EVs samples is the change in size as well as the affinity of real and synthetic CD-81 tetraspanin/peptide to CD-81 antibody.

As can also be deduced from these results, electrochemical label-free biosensors offered better sensitivity and limit of detection compared to standard ELISA technique. Moreover, DPV based detection seemed to be more promising in terms of providing more sensitive analysis.

### Biosensor selectivity

**Figure 6:**
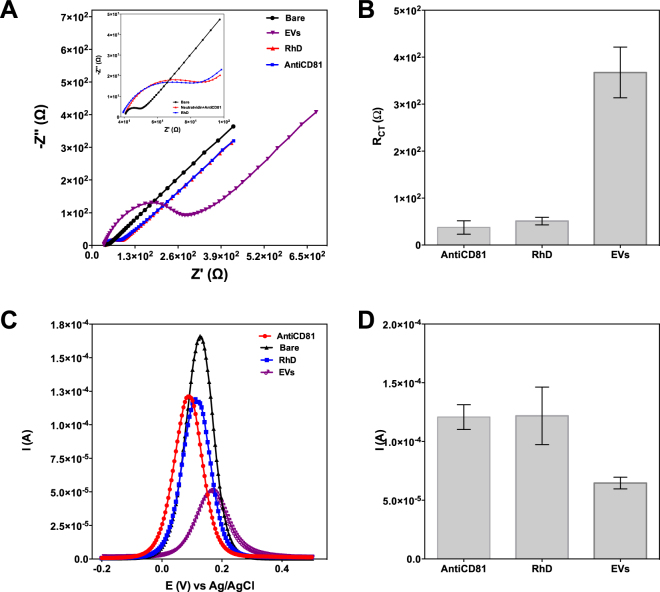
Results of the biosensor selectivity study showing the effect of RhD immobilization on Anti-CD81 Ab immobilized electrodes. Nyquist plots (**A**) and bar graphs representing R_ct_ values calculated from simulation results (**B**). Differential pulse voltammogram (**B**), and bar graphs representing Ip_A_ values. Data are expressed as mean ± standard deviation of three measurements. Experimental steps are detailed in methods section.

The assesment of the biosensor selectivity was done via control studies where a control protein was immobilized on Anti-CD-81 Ab immobilized Au-SPEs. We have chosen RhD as control since it is known to be abundant in blood as an erythrocyte membrane protein. To be sure that there are no non-specific binding-related false signals, a high protein concentration of RhD was chosen and tested on the developed biosensors. Control study results are represented by Fig. 6. We have performed the same experimental steps to functionalize AuSPE surface with antiCD-81 antibody and instead of immobilizing EVs, RhD solutions were drop casted and incubated on functionalized electrode surface. As it can be clearly seen by Fig. 6A, RhD does not impede any changes on the R_ct_ value compared to antiCD-81 antibody (Fig. 6A inset shows more clearly almost overlapping Nyquist plots of RhD and AntiCD81exposed Au SPEs) whereas when even a lower concentration of EVs were used as a sample, the Rct value was increased to 10-fold. Similarly, differential pulse voltammograms shown by Fig. 6C depicts the striking difference between EVs and RhD in terms of their effect on both oxidation peak current and potential. RhD did not affect neither the peak current nor the potential of Fe(CN)_6_]^3/4^ in a significant extent. However, voltammograms recorded for EVs detection showed a different tendency since the peak potential were shifted to more positive values and peak current were reduced to 6-fold compared to the value recorded for antiCD-81 modified AuSPE. Therefore, we conclude that, our sensor showed a very good selectivity towards CD81.

### Effect of CoCl_2_-induced hypoxia on the proliferation of the MCF-7 cell line

As it is reported by Wu *et al*., hypoxia can be induced via addition of 100 μM CoCl_2_ to growth media of MCF-7 cells^37^ on the second day of incubation. Exposure to CoCl_2_ leads to stabilization of hypoxia inducible factors such as HIF1-α, which promote the expression of genes related with oxygen transport, when binding to its promoter. During hypoxia, hypoxic signalling is activated by dimethyloxalylglycine and as one of the HIF family isoforms, HIF1-α is stabilized to regulate the genes that are involved in oxygen transport^38^. Hence, to screen hypoxia, HIF1-α is routinely detected.

**Figure 7:**
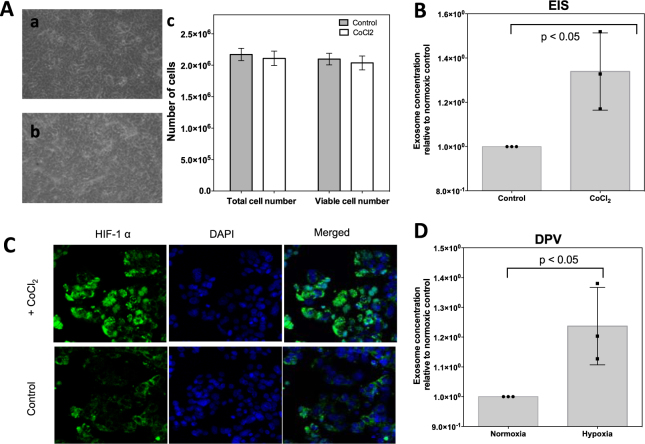
Effect of CoCl_2_-induced hypoxia on MCF-7 cell viability, HIF-1 alpha expression and EVs secretion. Effect of CoCl_2_ on cell viability; microscope images shows the cell morphology after (**A-a**) and before (**A-b**) CoCl_2_ exposure for incubation period of 3 days. Statistical analysis of total and viable cell numbers before and after CoCl_2_ exposure (**A-c**). Assessment of HIF1-α expression (in green) after exposure to CoCl_2_. DAPI was used as a counter stain (**D**), DPV results presented via voltammograms (**B**) and bar graphs representing normalized IpA (**C**), EIS results presented via Nyquist plots (**E**) and bar graphs representing normalized R_ct_ (**F**). Data are expressed as mean ± SD of three repeats.Experimental steps are detailed in methods section. Annotations * correspond to differences with *P* values < 0.05.

After exposure to hypoxic conditions for 24 hours, the cell culture medium was processed through ultracentrifugation and its concentration was obtained through a NTA analysis. The absolute number of EVs was normalized to the live cell number and NTA results showed higher EVs in the case of hypoxic condition. MCF-7 cells showed similar morphology both in hypoxia and normoxia as shown in Fig. 7A-a and Fig. 7A-b respectively. Total and viable cell counts represented by bar graphs in Fig. 7A-c prove that CoCl_2_ exposure doesn’t affect cell viability for the experimental duration (3 days). Expression of HIF-α was assessed through immunohistochemistry. Increased nuclear localization of HIF1-α upon CoCl_2_ exposure confirms the induction of hypoxia (Fig. 7D).

In previous studies, it has been shown that, hypoxic signalling activation results in significant increase in EVs release in breast cancer cells^38^. Therefore, the aim of this study was to quantify EVs by designed biosensors for normoxia and hypoxia conditions. Both DPV and EIS results indicate a significant change in exosome secretion after exposure to hypoxia. Figure 7B shows the exosome concentration relative to normoxic control based on DPV data and calibration curve. The ratio between exosome secretion levels was calculated as 1.23. Similarly, Fig. 7D shows the normalized exosome concentration with respect to normoxic control and has the exact same ratio of 1.23. The reported exosome secretion change with CoCl_2_ induced hypoxia was 1.77-fold increase with NTA analysis from MCF-7 cells^38^. Therefore the experimental findings support the literature.

## Discussion

This paper describes a label-free electrochemical detection method for EVs based on the transmembrane CD-81 biomarker. The designed biosensor neither requires any expensive microfabrication steps nor lengthy experimental steps. Unlike ELISA, a lengthy procedure that does not provide reproducible results, and has a very high LOD (2.84 × 10^14^ EVs/ml), our sensor enables a sensitive detection of EVs with a LOD of 77 EVs/ml with a RSD% smaller than 9.6%. Moreover, the reported LOD of the biosensor is lower than any biosensors reported for EVs detection. We also demonstrated the selectivity of the biosensor for CD-81 by using a blood-abundant protein, RhD as control considering the future applications of the sensor on detection of EVs inside blood.

In this study, we have also demonstrated for the first time in literature that, this label-free, low-cost biosensor based on commercially available, disposable screen printed electrodes can be used for detection of CoCl_2_ induced hypoxia triggered enhanced EVs secretion. MCF-7 cell line was used as a model breast cancer cell line and hypoxic conditions were maintained for three days. The exosome secretion level was calculated to be increased by 1.23-fold after CoCl_2_ exposure compared to normoxic conditions. The proposed biosensor does not require any labelling procedures, expensive microfabrication techniques and nanoparticle functionalization, therefore provides a very simple strategy with a superior limit of detection. Therefore, we believe that, this label-free biosensor has the potential to be integrated with cell culture platforms to monitor changes in EVs secretion triggered by relevant parameters such as oxygen tension.
